# DARPP-32 expression arises after a phase of dysplasia in oesophageal squamous cell carcinoma

**DOI:** 10.1038/sj.bjc.6601899

**Published:** 2004-06-08

**Authors:** Y Ebihara, M Miyamoto, A Fukunaga, K Kato, T Shichinohe, Y Kawarada, T Kurokawa, Y Cho, S Murakami, H Uehara, H Kaneko, H Hashimoto, Y Murakami, T Itoh, S Okushiba, S Kondo, H Katoh

**Affiliations:** 1 Surgical Oncology, Cancer Medicine, Division of Cancer Medicine, Hokkaido University Graduate School of Medicine, N15W7 Kita-ku, Sapporo, Hokkaido 060-8638, Japan; 2 Department of Pathology, Hokkaido University Hospital, N14W7 Kita-ku, Sapporo, Hokkaido 060-8648, Japan

**Keywords:** DARPP-32, oesophageal squamous cell carcinoma, immunohistochemistry, carcinogenesis, prognosis

## Abstract

This is the first report to correlate DARPP-32 immunoreactivity (dopamine and cAMP-regulated phosphoprotein, *M*_r_ 32 000) to clinicopathological status in human cancer. DARPP-32 is recognised as a neuronal protein. A recent study demonstrated that DARPP-32, and a truncated isoform t-DARPP, are overexpressed in gastric carcinoma during the process of carcinogenesis. The biological function of DARPP-32, however, is still unclear. The purpose of this study was to clarify the roles of DARPP-32 and t-DARPP in oesophageal squamous cell carcinoma (OSCC). Initially, we investigated DARPP-32 and t-DARPP expression in OSCC cell lines by Reverse transcription–polymerase chain reaction and Western blot. DARPP-32 expression was observed in four out of seven (57.1%) cell lines, but t-DARPP expression was not observed in any cell lines. In oesophageal tissue sample, DARPP-32 expression was observed in four out of seven (57.1%) tumour tissues, while t-DARPP was not observed in any tissues. Subsequently, DARPP expression was assessed by immunohistochemistry, using a polyclonal antibody, in tissue sections from 122 patients with primary OSCC. DARPP immunoreactivity was not observed in any normal oesophageal mucous membranes. On the other hand, positive DARPP immunostaining was detected in 37 patients (30.3%) and correlated inversely with pathologic stage (*P*=0.0284), pT (*P*=0.0438), pN (*P*=0.0303) and tumour size (*P*=0.012). The overall survival rate was worse in patients with DARPP-negative tumours than in patients with DARPP-positive tumours (*P*=0.0453). Interestingly, DARPP expression was observed in only one out of 45 cases of dysplasia. These observations suggest that DARPP-32 (rather than t-DARPP) expression arises after a phase of dysplasia in OSCC, and that tumours expressing DARPP-32 progress less rapidly than DARPP-32-negative tumours.

Oesophageal carcinoma remains a disease with poor prognosis. Advances in surgical technique and perioperative management have improved survival to some extent. The overall 5-year survival rate, however, generally remains less than 50%, even with the use of multimodality therapy (Ando *et al*, 1997, 2000; Collard *et al*, 2001). This is despite a better understanding of the molecular basis of oesophageal carcinogenesis and identification of prognostically important biologic markers.

DARPP-32 (dopamine and cAMP-regulated phosphoprotein, *M*_r_ 32 000) is recognised as a neuronal protein. In brain, DARPP-32 is a potent inhibitor of a cell cycle regulatory enzyme, protein phosphatase-1 (PP1) (Bibb *et al* 1999; Greengard, 2001), and can mediate phosphorylation and activation of mitogen-activated protein kinase (MAPK) and c-AMP-responsive element-binding protein (CREB), an inhibitor of apotosis (Yan *et al*, 1999). Furthermore, DARPP-32 orchestrates the degree of phosphorylation in a variety of molecular targets in the cell membrane and cytoplasm (Greengard, 2001). DARPP-32 and t-DARPP have been proposed to be factors providing an important survival advantage to neoplastic cells and may be important for gastric tumorigenesis (Wa'el *et al*, 2002). However, thus far, little is known about the role of DARPPs in carcinogenesis. Here, we examined DARPP-32 and t-DARPP expression and immunoreactivity in OSCC cell lines in archival material obtained from 122 surgical specimens of oesophageal squamous cell carcinoma (OSCC) with clinical and histopathologic factors obtained by a retrospective review of patient records.

## MATERIALS AND METHODS

### Cell lines and culture conditions

Human OSCC cell lines TE2, TE5, TE8, TE10 and TE13 were generously provided by Dr Nishihira (University of Tohoku, Japan). HEC46 was provided by Dr Toge (University of Hiroshima, Japan), and SGF7 was provided by Dr Saito (Toyama Medical and Pharmaceutical University, Japan). TE2, TE5, TE8, TE10, TE13 and HEC46 cells were grown in Dulbecco's modified Eagle's medium (D-MEM, Sigma-Aldrich Co., Ltd., Irvine, CA, USA) with 10% fetal bovine serum (FBS), and 1% penicillin/streptomycin (p/s). SGF7 cells were maintained in RPMI-1640 medium (Sigma-Aldrich Co., Ltd.) with 10% FBS and 1% p/s. All cell lines were maintained in a humidified incubator with 5% CO_2_ in air at 37°C.

### Tissue samples

Tumour and normal tissue samples were snap-frozen and stored at −80°C. The samples were obtained in 2002 from resections directly after surgery. Frozen sections (5 *μ*m) were stained with haematoxylin and eosin (H & E) to verify OSCC and approximately 0.1 g of the tumour block was processed for RNA extraction using the TRIZOL Reagent (GIBCO BRL, Grand Island, NE, USA).

### Reverse transcriptase–polymerase chain reaction (RT–PCR)

Total cellular RNA was isolated with TRIZOL Reagent (GIBCO BRL) from each cell line. Each 20 *μ*l cDNA synthesis reaction contained 1 *μ*g of total RNA, 1 × First Strand Buffer (GIBCO BRL); 50 mM Tris-HCL, pH 8.3, 75 mM KCL, 3 mM MgCl_2_), 0.5 mM of each deoxynucleotide triphosphate, 200 U of SUPERSCRIPT II (GIBCO BRL), 10 mM dithiothreitol and 0.5 *μ*g oligo (dT) (GIBCO BRL). The reverse transcription (RT) reaction was carried out for 50 min at 42°C and inactivated by heating at 70°C for 15 min. Multiplex PCR was performed as described previously (Wong *et al*, 1994). Briefly, each 25 *μ*l reaction contained 2 *μ*l of RT reaction products, 1 U of *Taq* DNA polymerase (Boehringer Mannheim, Germany), 1 × PCR buffer (Boehringer Mannheim), 160 mM of each deoxynucleotide and 20 pmol of each 3′ and 5′ primer specific for DARPP-32 (sense, 5′-gaagatccagttctcgg-3′; antisense 5′-ACTTAGTGCTGGGTCTTCC-3′), t-DARPP (sense, 5′-gttccggctctcagagca-3′; antisense 5′-ACTTAGTGCTGGGTCTTCC-3′), and *β-*actin (sense, 5′-AATCGTGCGTGACATTTAG-3′; antisense 5′-GTCCACGTCACACTTCATG-3′). DARPP-32, t-DARPP and *β*-actin cDNA were amplified for 30 cycles. Conditions for DARPP-32 PCR were 94°C for 30 s, 52°C for 30 s, then 72°C for 30 s. Conditions for t-DARPP PCR were 94°C for 30 s, 53°C for 30 s, then 72°C for 30 s. Conditions for *β*-actin PCR were 94°C for 30 s, 51°C for 30 s, then 72°C for 30 s. All PCR products were electrophoresed in a 2.0% agarose gel and visualised by ethidium bromide staining. As positive controls, plasmids expressing DARPP-32 or t-DARPP were generated by PCR amplification of the full-length cDNA derived from gastric cancer tissue and cloning into the *Bam*HI and *Hind*III sites of pCEP4 (Invitrogen Corp., Carlsbad, CA, USA).

### Western blot

Western blot analysis was performed to analyse DARPP-32 and t-DARPP expression in oesophageal cancer cell lines. Cell lysates were prepared in SDS buffer containing 62.5 mM Tris-HCl pH6.8, 2% w v^−1^ SDS, 10% glycerol, 50 mM DTT, 0.1% w v^−1^ bromphenol blue, 1 mM PMSF. Total proteins (40 *μ*g: OSCC cell lines, 2 *μ*g: tranfected cell lines) were electrophoresed in 15% SDS–polyacrylamide gels and transferred onto nitrocellulose membranes. Rabbit anti-DARPP polyclonal antibody (H-62; Santa Cruz Biotechnology, Santa Cruz, CA, USA) was used as the primary antibody (1 : 1000). Peroxidase-conjugated goat F(ab')_2_ anti-rabbit IgG (Jackson ImmunoReserch, West Grove, PA, USA) was used as the secondary antibody (1 : 5000). Detection of bound antibodies was performed using the ECL system (Amersham, Aylesbury, UK). The plasmids expressing DARPP-32 or t-DARPP were transfected into TE8 cells using Lipofectamine (Invitrogen Corp.) and these lysates were used as positive controls.

### Patients and oesophageal specimens

All complete OSCC surgical specimens resected from 1989 to 1999, from patients with no evidence of metastasis to other organs and without prior anticancer treatment, were examined. Cases of in-hospital death were excluded. Surgical specimens from 122 patients who had undergone radical oesophagectomy at the Department of Surgical Oncology, Hokkaido University, Hokkaido Gastroenterology Hospital, and Teine Keijinkai Hospital were included in the current study, and findings were referred to the patients' clinical records. One of the sections from the deepest point of each tumour invasion was selected for evaluation. The specimens were examined histologically after staining with H & E, and the clinicopathologic stage was determined according to the TNM classification system of the International Union Against Cancer (Sobin and Wittekind, 2002). Specimens from 122 patients were included in the current study (105 males and 17 females). The median patient age was 62.3 years (range, 38–82 years). A relatively large number of patients had early-stage disease (78 patients, 63.9%). In total, 61 patients (50.0%) had lymph node metastases and 19 patients (16%) had distant nodal metastases. The study population had the following performance status (PS): PS0, 107 patients; PS1, 14 patients; and PS2, one patient. The median follow-up period was 29 months.

All specimens were fixed in 10% formalin and embedded in paraffin wax. One of the deepest sections from each tumour was selected for evaluation, and serial 4 *μ*m-thick sections were examined by immunohistochemistry.

### Immunohistochemistry

Unstained sections were treated with a rabbit anti-DARPP polyclonal antibody (H-62; Santa Cruz Biotechnology; 1 : 200 dilution), using previously described conditions (Oliver and Shenolikar, 1998). Briefly, each slide was deparaffinised by routine techniques, treated with sodium citrate buffer (Ventana-Bio Tek Solutions, Tucson, AZ, USA), and then treated with microwave heat for 20 min. After cooling for 5 min, slides were labeled with antibody using the Ventana EX system automated stainer (Ventana-Bio Tek Solutions). The anti-DARPP-32 antibody was detected by adding biotinylated secondary antibody, avidin–biotin complex and 3, 3′-diaminobenzene (Ventana DAB Universal Kit; Ventana-Bio Tek Solutions). Sections were then counterstained in haematoxylin for 1 min and mounted in Permount (Microslides; Muto-Glass, Tokyo, Japan). For a negative control, nonimmune purified rabbit serum was used for the primary antibody. The number of stained cells per 1000 was determined under a microscope (Olympus Optical Co., Ltd, Tokyo, Japan) in three visual fields, at a magnification × 200. When microscopic examination indicated that a the total number of cancer cells observed being less than 1000, all cells were counted. When over 10% cancer cell nuclei and cytoplasm were stained, the tumour was considered DARPP-32 positive. The current study was performed in a retrospective manner, while all specimens were evaluated by three investigators (YE, MM and TI), who were blinded to the patients' clinical information. Dysplasia was defined according to the WHO classification (Stanley and Lauri, 2000).

### Statistical analysis

Either the *χ*^2^ test or Fisher's exact test were used to analyse the correlation between DARPP-32 expression and patients' parameters, including histopathologic findings. The Kaplan–Meier method was used to generate survival curves, and survival differences were analysed with the log-rank test, based on the status of DARPP-32 expression. Univariate and multivariate analyses of DARPP immunoreactivity and clinicopathological features were performed using the Cox proportional hazard regression model. Probability values of less than 0.05 were regarded as indicating significance. All analyses were performed using statistical analysis software (Statview J version 5.0; SAS Institute Inc., Cary, NC, USA).

## RESULTS

### Expression of DARPPs in OSCC cell lines

**Figure 1:**
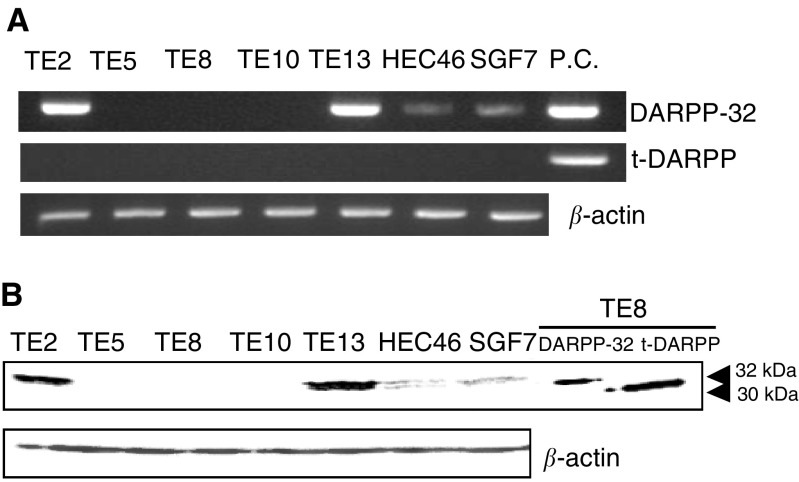
RT–PCR expression analyses. (**A**) A gel imaging for DARPP-32, t-DARPP and *β*-actin expression at 30 cycles by RT–PCR. Seven oesophageal squamous cell lines, and positive controls (pCEP4-DARPP-32, pCEP4-t-DARPP) are shown. (**B**) DARPP protein expression. Seven oesophageal squamous cell lines and TE8 cells transfected with pCEP4-DARPP and pCEP4-t-DARPP were separated by SDS–PAGE and analysed for DARPP protein expression by Western blot with a COOH-terminal DARPP-32 antibody (Santa Cruz Biotechnology).

DARPP-32 RT–PCR fragments were amplified from four out of seven cell lines, while t-DARPP fragments were not amplified in any cell lines (Figure 1A

). Western blot analysis showed that a 32 kDa protein, corresponding to DARPP-32, was expressed strongly in TE2 and TE13, while t-DARPP protein (30 kDa) was not detectable in any cell lines, as expected based on the RT–PCR data (Figure 1B).

### Detection of DARPPs in human tissues

**Figure 2:**
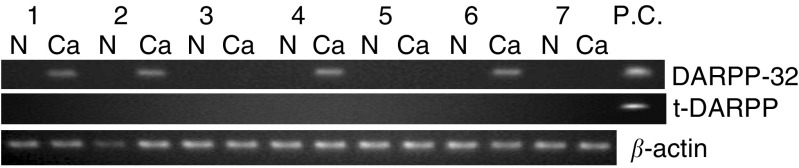
RT–PCR expression analyses. A gel imaging for DARPP-32, t-DARPP and *β*-actin expression at 30 cycles by RT–PCR. Seven different samples of normal oesophageal mucosa (N), tumour tissues (Ca) and positive control (pCEP4-DARPP-32, pCEP4-t-DARPP) are shown.

In oesophageal sample tissues, DARPP-32 RT–PCR fragments were amplified from four out of seven tumour tissues, but not from any normal oesophageal mucosa. As in the cell lines, t-DARPP RT–PCR fragments were not amplified from any sample tissues (Figure 2

).

### Immunohistochemical analysis

**Figure 3:**
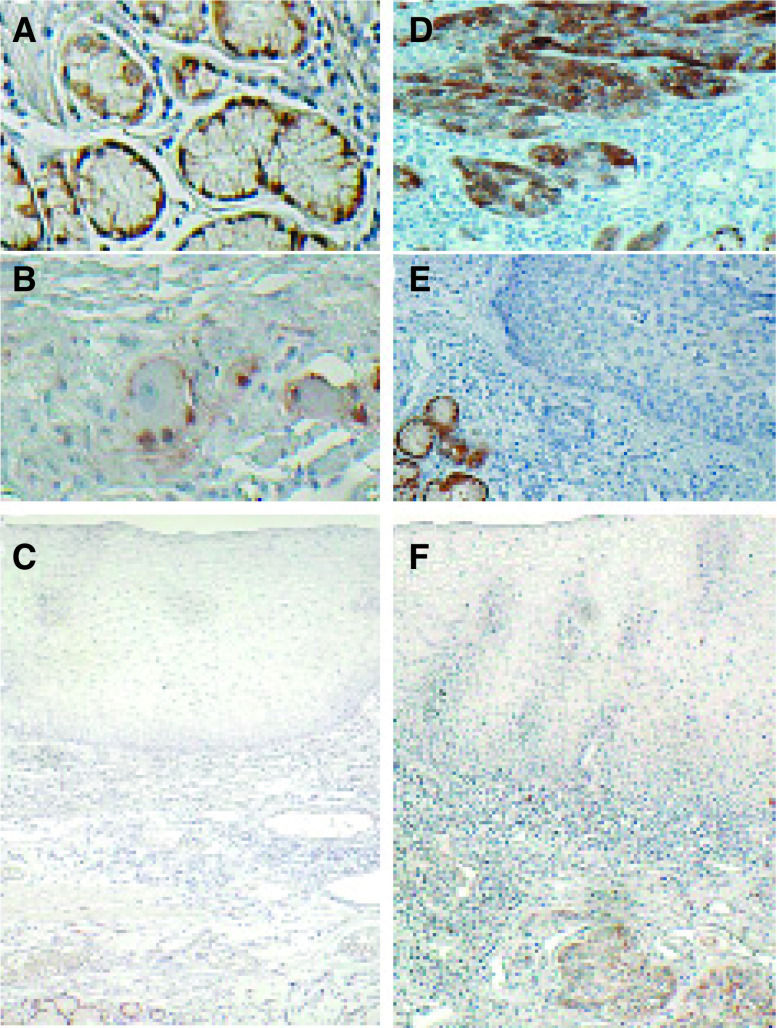
Immunohistochemical staining for DARPP. (**A**) Oesophageal gland cells, (**B**) gangliocytes stained as an internal control. (**C**) DARPP expression is not detected in normal oesophageal mucous membrane. (**D**) Almost all the cancer cell membrane and cytoplasm were stained for DARPP. (**E**) DARPP immunoreactivity was not observed in cancer cells, while gland cells showed strong staining as an internal control. (**F**) DARPP immunoreactivity was not observed in dysplastic cells, while cancer cells showed staining. (original magnifications: **A**, **B**, × 400; **C**, × 40; **D**, **E**, × 100; **F**, × 40).

We subsequently performed immunohistochemical analysis on the 122 OSCC specimens. This analysis provides suggestions as to the biological function of DARPP in OSCC. DARPP immunoreactivity was observed at the cancer cell membrane and cytoplasm, as seen in a previous study (Wa'el *et al*, 2002). DARPP was expressed in normal oesophageal gland cells (Figure 3A

) and gangliocytes (Figure 3B), permitting their use as a positive internal control. No DARPP expression was detected in normal oesophageal mucous membrane (Figure 3C). In tumour cells, immunoreactivity was observed very vividly (Figure 3D), with 37 patients (30.3%) staining positive for DARPP and 85 patients (69.7%) staining negative (Figure 3E).

Dysplasias were found in 45 of 122 specimens, but DARPP expression was only observed in one of theses lesions. A total of 36 patients with DARPP-positive tumours had DARPP-negative dysplasias (Figure 3F).

### Statistical analyses between DARRP expression and clinicopathological data

DARPP immunoreactivity had an inverse relationship with pathologic stage, pT, pN and tumour size by the *χ*^2^ test (Table 1

**Table 1:** Relationship between clinicopathologic features and DARPP-32 expression in surgical specimens of oesophageal squamous cell carcinoma

**Variables**	**DARPP-32 positive (*n*=37)**	**DARPP-32 negative (*n*=85)**	***P* value^a^**
Gender			
Male	32	73	0.9294
Female	5	12	
			
Age			
⩾65	11	38	0.1209
<65	26	47	
			
p-Stage			
I, II	29	49	0.0284
III, IV	8	36	
			
Grade			
G1	11	20	0.4696
Others	26	65	
			
p-T classification			
T1, T2	26	43	0.0438
T3, T4	11	42	
			
p-N classification			
N0	24	37	0.0303
N1	13	48	
			
p-M classification			
M0	31	72	0.8973
M1	6	13	
			
Tumour size			
⩾4.5 cm	13	51	0.0115
<4.5 cm	24	34	
			
Surgical margin			
Positive	2	6	0.7345
Negative	35	79	
			
Adjuvant therapy			
Yes	17	33	0.4622
No	20	52	

a The *P*-value was calculated by chi-square test.

). Moreover, overall survival rate by the Kaplan–Meier method was worse in patients with DARPP-32-negative tumours than in patients with DARPP-32-positive tumours (Figure 4

**Figure 4:**
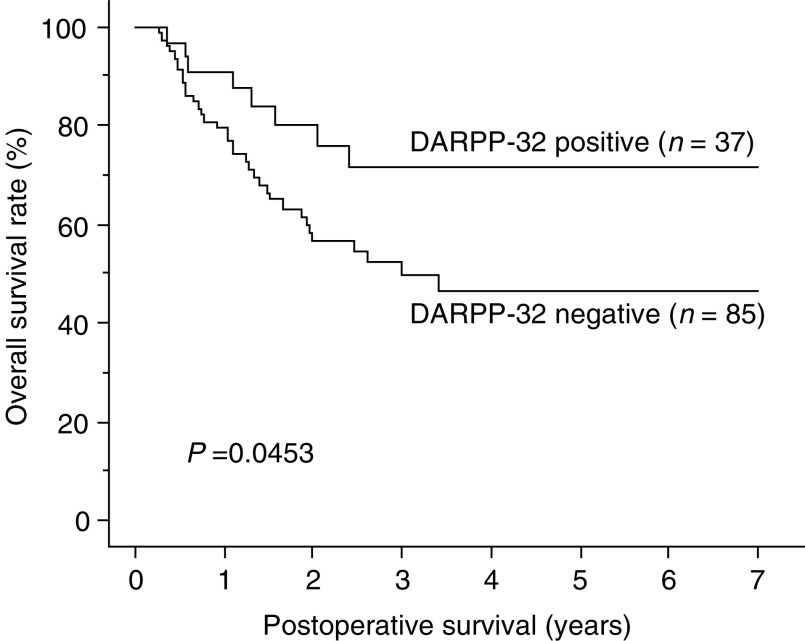
Comparison of overall survival curves for patients with DARPP-32-positive and -negative tumors out of 122 patients who underwent radical oesophagectomy.

). Upon univariate analysis with Cox proportional hazards model, DARPP immunopositivity was inversely correlated with poor prognosis, although, multivariate analyses did not indicate that DARPP positivity was significant (data not shown).

## DISCUSSION

DARPPs are frequently expressed in gastric cancer. To assess the biological significance, we screened for DARPP expression in several types of cancer cell lines. Interestingly, we found that there were two types of OSCC cell lines, DARPP-expressing and -nonexpressing cells.

The RT–PCR results appear to accurately reflect the status of DARPP expression. Although both DARPP isoforms were frequently overexpressed in gastric cancer, 30% of OSCC samples expressed DARPP-32 alone. Based on our RT–PCR and Western blot data, we believe that DARPP immunoreactivity in OSCC specimens is specific to DARPP-32 but not t-DARPP.

In TE2 and TE13 cell lines, manual sequencing analyses were performed, but no genetic alterations of DARPP-32 were found (figure not shown). Therefore, the difference in DARPP-32 expression is not likely to be caused by mutation.

Several reports have described the pattern of progression from normal mucous to dysplasia, to carcinoma *in situ* (Shi *et al*, 1999; Saeki *et al*, 2002; Shirai *et al*, 2002). Oesophageal dysplasia is also believed to be one of the precursors for OSCC. The ratio of DARPP immunopositivity in dysplasia was significantly lower (*P*<0.0001; *χ*^2^ test) compared to that in tumour. These data suggest that overexpression of DARPP arises at the late phase of neoplastic progression of the oesophagus. Moreover, these features could be an advantage to distinguish between cancer and dysplasia in diagnosis of biopsy specimens (30.3% sensitivity, 97.8% specificity).

It remains unclear whether DARPP-32 is an oncogene like ras, myc and src because some normal tissue expresses DARPP-32. Overexpression of DARPP-32, however, is related to carcinogenesis in 30% of OSCC. Moreover, DARPP-32-positive tumours appear to have a less aggressive character than DARPP-32 negative tumours. This characteristic is not due to exclusion by the host immune response. We previously reported that the cooperative role of CD4^+^ and CD8^+^ T cells appears to drastically improve the prognosis of patients with OSCC (Cho *et al*, 2003). Despite the better prognosis in patients with DARPP-32-positive tumours, the expression of DARPP-32 in OSCC was not correlated with infiltration of CD4^+^ and/or CD8^+^ T cells (*P*=0.7409; *χ*^2^ test).

DARPP-32 is a known protein that acts as a PP1 inhibitor or an MAPK, CREB mediator. Protein phosphatase 1 and other protein phosphatases that reverse the action of cyclin-dependent kinases are emerging as important cell cycle regulatory enzymes (Oliver and Shenolikar, 1998; Schonthal, 2001; Wang *et al*, 2001). Many oncogenes have been shown to encode proteins that trasmit mitogenic signals upstream of the MAPK pathway (Seger and Krebs, 1995; Sebolt-Leopold, 2000). Phosphorylation of CREB generates signals that inhibit apotosis (Jean *et al*, 1998; Jean and Bar-Eli, 2000). Control of phosphorylation in gastrointestinal malignancies has recently been reported as an important mechanism for some neoplasia (Saha *et al*, 2001; Higashi *et al*, 2002). To understand the potential role of DARPP-32 in human OSCC, however, additional studies and biological assays are required. We are now proceeding with further investigation to clarify the biological function of DARPP-32 in OSCC cells.

We conclude that DARPP-32 expression arises after a phase of dysplasia in OSCC. Moreover, DARPP-32-positive tumours have a less aggressive character than those that are not.
